# Mechanism(s) of action of heavy metals to investigate the regulation of plastidic glucose-6-phosphate dehydrogenase

**DOI:** 10.1038/s41598-018-31348-y

**Published:** 2018-09-07

**Authors:** Alessia De Lillo, Manuela Cardi, Simone Landi, Sergio Esposito

**Affiliations:** Dipartimento di Biologia, Università di Napoli Federico II, Naples, Italy

## Abstract

The regulation of recombinant plastidic glucose-6P dehydrogenase from *Populus trichocarpa* (*Pt*P2-G6PDH - EC 1.1.1.49) was investigated by exposing wild type and mutagenized isoforms to heavy metals. Nickel and Cadmium caused a marked decrease in *Pt*P2-G6PDH WT activity, suggesting their poisoning effect on plant enzymes; Lead (Pb^++^) was substantially ineffective. Copper (Cu^++^) and Zinc (Zn^++^) exposition resulted in strongest decrease in enzyme activity, thus suggesting a physiological competition with Magnesium, a well-known activator of G6PDH activity. Kinetic analyses confirmed a competitive inhibition by Copper, and a mixed inhibition by (Cd^++^). Mutagenized enzymes were differently affected by HMs: the reduction of disulfide (C^175^–C^183^) exposed the NADP^+^ binding sites to metals; C^145^ participates to NADP^+^ cofactor binding; C^194^ and C^242^ are proposed to play a role in the regulation of NADP^+^/NADPH binding. Copper (and possibly Zinc) is able to occupy competitively Magnesium (Mg^++^) sites and/or bind to NADP^+^, resulting in a reduced access of NADP^+^ sites on the enzyme. Hence, heavy metals could be used to describe specific roles of cysteine residues present in the primary protein sequence; these results are discussed to define the biochemical mechanism(s) of inhibition of plant plastidic G6PDH.

## Introduction

In higher plants the oxidative pentose phosphate pathway (OPPP) plays multiple and fundamental roles, such as the synthesis of precursors for nucleic acids and fatty acids^1^; the furnishing of NADPH essential for primary metabolism^2,3^, and to counteract oxidative stress^4–6^.

It is well known that a pivotal role in the whole process is played by glucose-6-phosphate dehydrogenase (G6PDH; EC 1.1.1.49), by catalyzing the conversion of glucose-6-phosphate to 6-phosphogluconate in the presence of NADP^+^^1^. The possible regulatory role of Magnesium (Mg^++^) in plant G6PDHs has been suggested^7^. Previously, the dependence on G6PDH reaction from Mg^++^ has been initially described in Bacteria^7,8^; moreover, it was suggested that Mg^++^ is able to affect the oligomeric state of the enzyme in dog liver G6PDH^9^ and human placental G6PDH^10^.

The existence of cytosolic and plastidic OPPPs in plants has been reported in plants^11^ based on the presence of cytosolic (Cy-G6PDH), chloroplastic (P1-G6PDH), and plastidic (P2-G6PDH) isoforms of the enzyme^1,2,12^.

Particularly, plastidic P2-G6PDH transcripts are detectable throughout the plant, more abundantly in stems and roots^13–15^. Namely in the root tissues, P2-G6PDH plays a pivotal role in providing reductants for anaplerotic metabolism, during nitrogen assimilation in non-photosynthetic plastids^2,16^, and fatty acid synthesis^17^.

G6PDHs from living organisms have been characterized from a number of sources, and kinetic properties of plant plastidic isoforms have also been described previously^12,14,18–21^.

G6PDH activity is generally increased during plant exposition to various biotic^22^ and abiotic stress^4,5,23,24^. Dal Santo *et al*.^25^ suggested that NADP^+^ binding is favored by phosphorylation of Thr^467^ by glycogen synthase kinase 3 in *Arabidopsis* (ASKα), thus increasing stability and activity of *A.thaliana* cytosolic G6PDH under oxidative stress conditions^25^. In plants, plastidic G6PDHs present two cysteines involved in a regulatory disulfide^20,26^. Née *et al*.^26^ demonstrated that, in chloroplastic P1-G6PDH, the formation of the regulatory disulfide bridge influences Arg^131^ position and NADP^+^ correct positioning. The comparison with human G6PDH suggests that this loop “guards” the access to NADP^+^ cofactor molecule to the active site, and possibly would be able to regulate G6PDH activity^15,21^. Interestingly, *Pt*P2-G6PDH mutants in the regulatory cysteines (C175-C183^21^) lack of redox regulation and show a low activity. C145 is located near the loop and could play a role in its orientation and/or stability: C145S mutants showed a similar behavior to WT enzyme, presenting a mixed inhibition by NADPH^21^. The other two cysteines are located sufficiently far from the active site, and this possibly explain the loss of NADPH inhibition in these mutants^21^.

Among abiotic stresses, heavy metals (HMs) are of greater concern due to their persistence in the environment, and the possibility to be absorbed/accumulated in living organisms^27,28^ and particularly in plants^29^. Different HMs have been suggested to induce changes in G6PDH activities in higher organisms such as fishes^30–32^, Anfibia^33^, Mammalia^34^ and Planta^35–37^.

Generally, HMs play important roles in plant metabolism: some of them are essential micronutrients, but some others represent highly toxic plant pollutants. In photosynthetic organisms HMs can be adsorbed both from atmospheric fallout caused by anthropic emissions^38^; or/and up-taken from waters polluted by industrial and urban waste^39^, inducing severe ultrastructural, physiological and biochemical damages.

The aim of this study is to utilize both essential and toxic heavy metals to investigate the changes in the activity and kinetic properties of purified recombinant plastidic isoforms from *Populus trichocarpa*. In order to establish the possible direct effect of specific HMs on enzymatic activity and regulatory properties, the recombinant, his-tagged plastidic *Pt*P2-G6PDH WT from *Populus trichocarpa* was incubated in the presence of HMs to establish the effect(s) of specific elements.

Moreover, mutagenized enzymes, lacking cysteine residues, were produced and tested for the possible interactions of HMs with disulfide bridges present in the active enzyme.

The results will be discussed in order to define the specific roles played by the cysteine residues present in the primary sequence in the regulation of plant G6PDH.

## Results

### Overexpression and purification of recombinant *Pt*P2-G6PDH

The recombinant *Pt*P2-G6PDH WT and cysteine- mutagenized enzymes were overexpressed and successfully purified^21^ (Supplementary Fig. S1), and kinetically characterized (Supplementary Table S1); then, the effects HMs on *Pt*P2-G6PDH activity were investigated. Furthermore, in order to test the possible effect of his-tag on enzymatic activity, *Pt*P2-G6PDH was alkylated (see Materials and Methods), and this enzyme preparation showed over 92% of activity with respect to the purified his-tagged *Pt*P2-G6PDH used throughout this work (not shown); therefore, we assumed that the his-tag did not affect the degree of inhibition by HMs.

### *In vitro* inhibition of *Pt*P2-G6PDH WT by heavy metals

**Figure 1:**
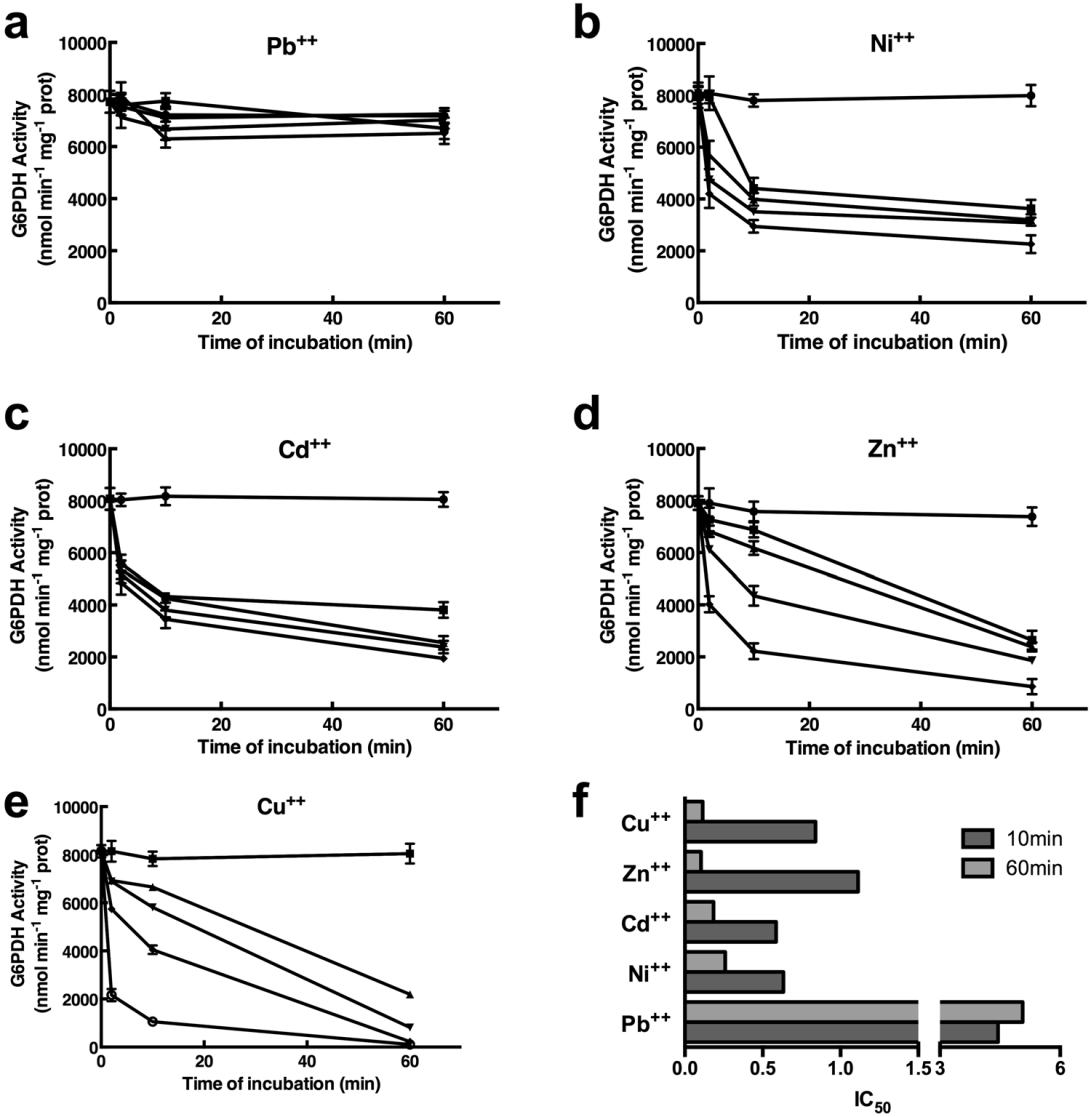
Effects of heavy metals on recombinant *Pt*P2-G6PDH WT. Purified enzyme was incubated in the presence of different metals at increasing concentrations for given times. (**a**) Lead (Pb^++^); (**b**) Nickel (Ni^++^); (**c**) Cadmium (Cd^++^); (**d**) Zinc (Zn^++^); (**e**) Copper (Cu^++^); (**f**) IC_50_ values (mM), calculated after 10 min (dark grey) and 60 min (light grey) of incubation with different HMs, related to *Pt*P2-G6PDH WT activity. Enzymatic rate was expressed as nmol • mg prot min^−1^. Symbols represent increasing heavy metals concentrations: (●) 0 mM; (■) 0.1 mM; (▲), 0.5 mM; (▼), 1 mM; (♦), 2 mM.

The recombinant purified *Pt*P2-G6PDH WT was incubated with different heavy metals: Cadmium (Cd^++^), Copper (Cu^++^), Zinc (Zn^++^), Nickel (Ni^++^), Lead (Pb^++^) were tested; assays were carried out under standard conditions by varying concentration of each metal from 0.1 mM to 2 mM at different times of incubation, from 2 min to 1 h (Fig. 1); further assays were made after 5 h and 10 h of incubation with heavy metals, resulting in slower decrease rate with respect to 1 h activity levels. Anyway, after 10 h of exposition to HMs the activities were 12–20% of unexposed controls; the only exception were Pb^++^-exposed enzymes, retaining more than 95% of activity after 10 h (see below). It should be underlined that no evident change was observed between sulfate and chloride salts of heavy metals, except for a 15–20% marked inhibition using ZnSO_4_ with respect with ZnCl_2_ (Supplementary Fig. S2).

Untreated *Pt*P2-G6PDH WT retained more than 98.5% of its initial activity up to 10 h.

Different metals affected G6PDH activity in a dose and time dependent mode. Intriguingly, Lead was the only element that did not substantially affect G6PDH rate (Fig. 1a): less than 8% inhibition was observed up to 10 h (not shown).

Ni^++^ and Cd^++^ effects were similar and independent from levels: after 10 minutes the inhibition was between 45–65%; after, a slower decrease was observed (<15% of the initial rate after 10 h - not shown) (Fig. 1b,c).

Zn^++^ caused a gradual decrease in function of the utilized concentrations and time. After 2 min there was a 25% decrease at 1 mM, and 50% at 2 mM; after 1 h the enzymatic activity was less than 25% of the starting activity (Fig. 1d). The Cu^++^ effect is gradual and, independently of the concentration, more drastic than Zn^++^: 1 mM Cu^++^ caused a 30% decrease in 2 min; after 1 h the residual activity was lower than 25%, independently from Cu^++^ concentration (Fig. 1e).

IC_50_ values for different HMs were calculated from nonlinear regression curves (Supplementary Fig. S3); as expected, IC_50_ for Pb^++^ was very high (about 5), denoting the ineffectiveness of Pb^++^ at different levels and incubation times on enzymatic activity.

The IC_50_ values calculated after 10 min confirmed that, at shorter incubation times, low concentrations of Cd^++^ or Ni^++^ led to stronger inhibition than Zn^++^ and Cu^++^ at the same levels; the sequence of inhibitory potentials of IC_50_ after 10 min was Cd^++^ > Ni^++^ > Cu^++^ > Zn^++^ > Pb^++^ (Fig. 1f).

Intriguingly, when IC_50_ values were calculated after 60 min of incubation, Zn^++^ and Cu^++^ resulted more effective with respect Cd^++^ and Ni^++^, confirming the gradual mechanism of inhibition played by the “physiological” cations; the “polluting” cations exerted a prompt and unspecific inhibition of the enzymatic activity, thus following the inhibitory sequence: Cu^++^ > Zn^++^ > Cd^++^ > Ni^++^ > Pb^++^ (Fig. 1f).

To check the possible his-tag influence on the HM inhibition, due to the potential binding of metals to the histidine residues of the tag, the alkylated enzyme was tested, and the corresponding IC_50_ calculated, confirming that the his-tag did not sensibly influence the HMs binding: all the values estimated on the alkylated protein were between 84% and 111% of the IC_50_s of the his-tagged enzyme, except for Cu^++^ (2.5 higher); anyway, Cu^++^ remained the more effective HM inhibitor on *Pt*P2-G6PDH WT (Supplementary Fig. S3f).

### Effects of Magnesium on *Pt*P2-G6PDH activity

In the previous experiments, the effects of HMs were measured in presence of saturating Mg^++^, a well-known activator of G6PDH activity. Thus, a fully desalted *Pt*P2-G6PDH WT was prepared in order to assess increase of the reaction rate at increasing Mg^++^ concentrations.

**Figure 2:**
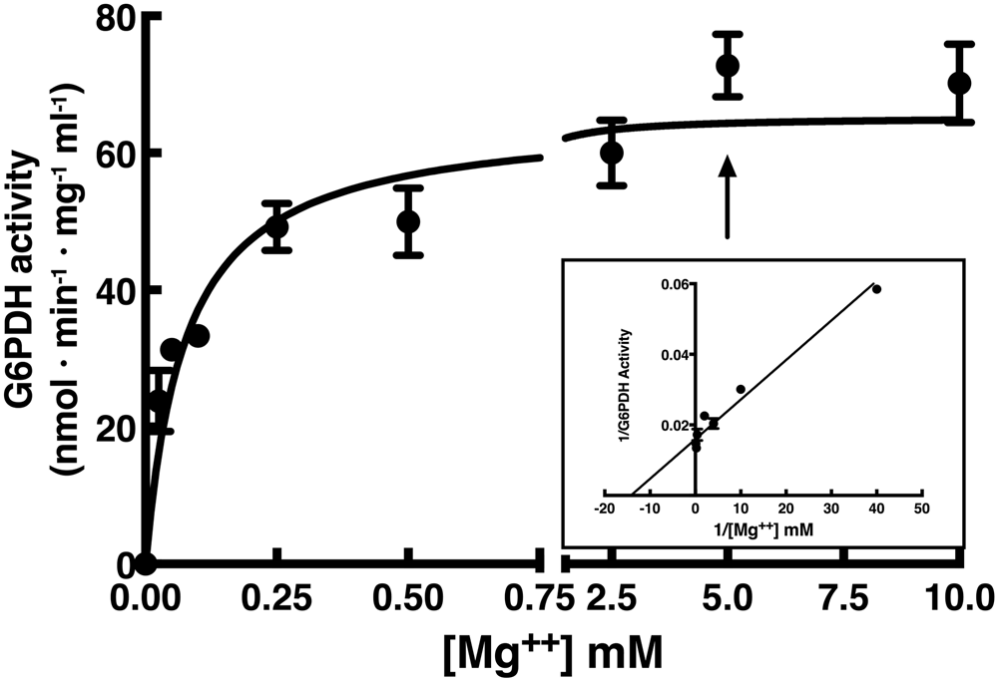
Saturation curve for Mg^++^ of purified recombinant *Pt*P2-G6PDH WT. Purified enzyme (IMAC step) was desalted twice in Sephadex G25 to eliminate all Mg^++^ (null activity). This preparation was used for the determination of Km_Mg_^++^ = 76 ± 13 μM. The insert shows the double reciprocal plot of the results. The arrow indicates the standard assay conditions.

The purified enzyme was sequentially desalted twice by gel filtration (Sephadex G25) until the enzymatic activity was substantially null. Then, an activation experiment by increasing Mg^++^, resulted in a Michaelis-Menten kinetic, exhibiting a Km_Mg_^++^ of 76 ± 13 μM (Fig. 2).

**Figure 3:**
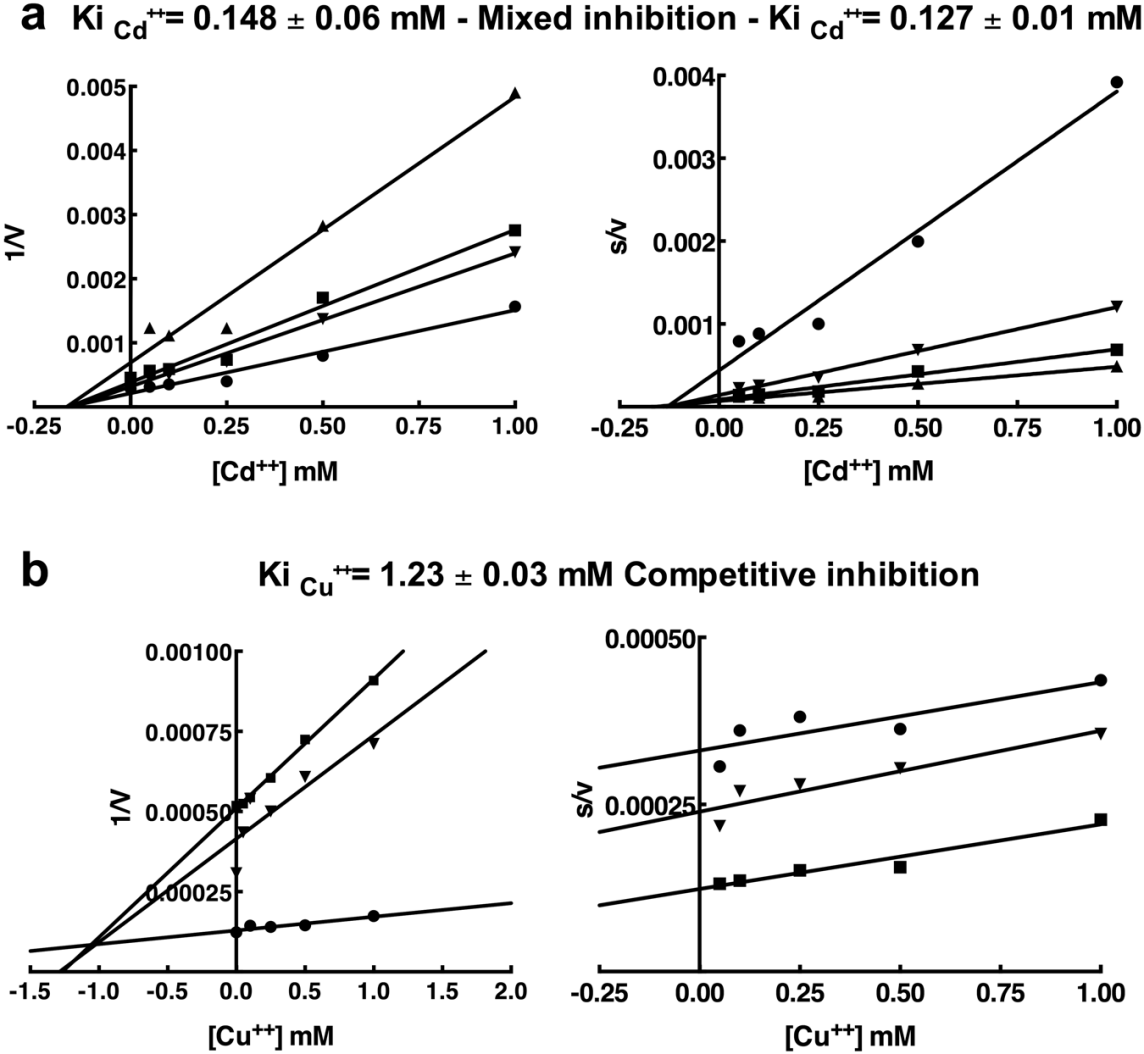
Determination of the type of inhibition and inhibition constant values of *Pt*P2-G6PDH WT exposed to Cadmium (Cd^++^) (**a**) and Copper (Cu^++^) (**b**) in the presence of different Mg^++^ concentrations. The description of the inhibition, Ki_Cd++_ and Ki_Cu++_ are indicated. On the left the Dixon plots; on the right the Cornish-Bowden plots. Different symbols indicate [Mg^++^] in the assay mixture: (▲), 0.1 mM; (■) 0.25 mM; (▼), 0.5 mM; (●) 2.5 mM. The regressions were calculated by Graphpad Prism software within 93% confidence.

The antagonism between the functional metal Mg^++^ and different heavy metals on the activation of enzymatic activity was tested in competition experiments. G6PDH was tested at different concentrations of Mg^++^ and increasing levels of HMs, in order to observe the possible competition with Mg^++^. As result, the inhibition caused by Cadmium and Copper is shown in Fig. 3, where both Dixon plots (1/V vs [inhibitor]) and Cornish-Bowden Plots (S/V vs [inhibitor]) are shown. The association of these plots is able to describe the type of the inhibition exerted by HMs^35^.

Interestingly, Cd^++^ inhibition can be described as of a mixed-type, resulting in low Kis, 0.148 mM and 0.127 mM; it should be noted that these values are 2-fold higher, but in the same order of magnitude as Km_Mg_^++^ (Fig. 3a).

Inhibition by Cu^++^ resulted in a Ki_Cu_^++^ 20-fold higher −1.23 mM - with respect to Km_Mg_^++^ (76 μM) (Fig. 3b); the double reciprocal plot of Km_Mg_^++^ under increasing levels of Cu^++^ suggests a competitive inhibition by Cu^++^ (Supplementary Fig. S4). The observed difference between the strong inhibition by Copper at 5 mM Mg^++^ (Fig. 1e), and the lighter effect at 2.5 mM Mg^++^ (Fig. 3b) can be possibly caused by the different times of competition between Mg^++^ and Cu^++^ on purified enzyme (minutes indicated on the x-axes in Fig. 1; and few seconds in Fig. 3e); and/or an inhibitory effect by Cu^++^ on fully activated enzyme (Mg^++^-stabilized form).

This competitive inhibition by Copper has been proved to be reversible at Cu^++^ concentrations lower or equal to 0.1 mM: *Pt*P2-G6PDH deprived of Mg^++^ was progressively reactivated by 0.5–5 mM Mg^++^, and the addition of Cu^++^ proportionally inhibited enzymatic activity (Supplemental Fig. S5A,B); when the desalted *Pt*P2-G6PDH was exposed to 0.01–0.1 mM Cu^++^, the addition of 0.5–5 mM Mg^++^ fully restored the activity (Supplemental Fig. S5C,D); in contrast, the inhibition by 1 mM Cu^++^ was irreversible (Supplemental Fig. S5E), confirming the effects of high levels of copper on enzymatic structure^40^: Cu^++^ is able to bind more tightly than Mg^++^, inactivating the enzyme.

Furthermore, by comparing the results shown in Supplemental Fig. S5B,C, it looks that the enzyme initially exposed to Mg^++^ (Fig. S5B) is more sensitive to Cu^++^ than the enzyme firstly exposed to Cu^++^ (Fig. S5C).

### Effects of heavy metals on cysteine mutagenized *Pt*P2-G6PDH

Five conserved cysteines present in the encoding *Pt*P2-G6PDH and other plastidic/chloroplastic isoforms have been previously identified (Supplementary Table S2); recombinant proteins with single cysteine substituted by serine were obtained. Analyses on these mutagenized *Pt*P2-G6PDH enzymes have been made at different time intervals and changing HMs concentrations as for WT protein, but the results did not add additional information (not shown) and will not further discussed here.

**Figure 4:**
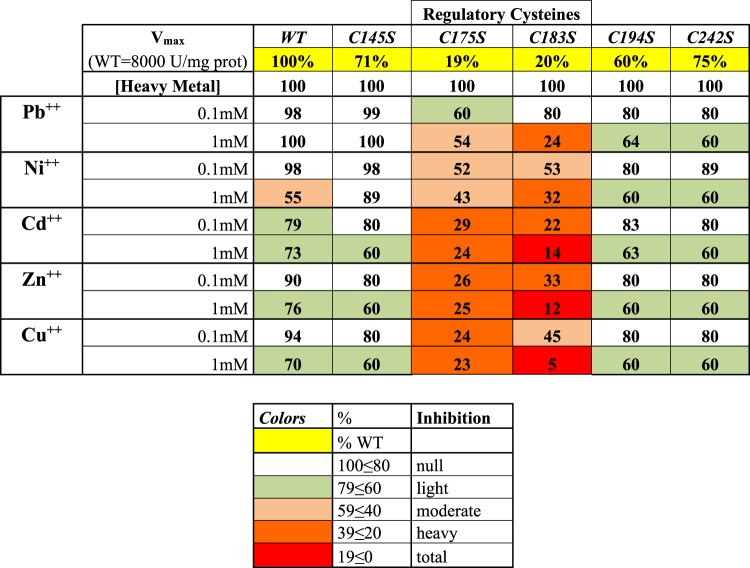
Effect of heavy metals on cysteine mutagenized *Pt*P2-G6PDH mutants. Mutagenized enzymes are indicated as CxxxS, where xxx is the residue number in the encoding sequence. For each element two metals’ concentrations were tested, 0.1 mM and 1 mM, and activities were assayed after 10 min of incubation. Results are reported as percentage of the maximum activity measured in unexposed samples. In the first row (yellow-highlighted line) maximum activity for each mutant is given as percentage of WT (V_max_ = 100% = 8000 U/mg prot). A color legend is provided to facilitate the reading of the results.

Based on the results obtained on *Pt*P2-G6PDH WT, we chose to test the inhibition rate at 0.1 mM and 1 mM HMs after 10 min of exposition. This time frame represents the interval of evident effect(s) of HMs, and it is assumed to be enough rapid to avoid severe secondary effects on mutagenized proteins, but not too fast to avoid the action of single HMs on the enzyme. Furthermore, the two concentrations selected represent the levels of HMs in the same order of Km_Mg_^++^ (76 μM), supposed to induce a physiological competition between Mg^++^ and other HMs; and one order of magnitude higher than Km (1 mM) simulating a toxic effect by metals. The results are displayed in Fig. 4.

Preliminarily, it should be observed that all cysteine-mutagenized proteins exhibited a V_max_ between 60 and 75% of WT, except for C175S and C183S, symbolizing the reduced, inhibited enzyme, with V_max_ < 20% of WT.

C145S mutant exhibited the same sensitivity to HMs observed for WT enzyme (Fig. 4). C194S and C242S showed a similar inhibition by all heavy metals tested, about 20% at 0.1 mM and 40% at 1 mM, denoting a general de-stabilization of the enzymes produced by heavy metals (Fig. 4). The recombinant enzymes mutagenized in the regulatory cysteines (C175S and C183S), even if showing lower activities - mimicking the enzyme with reduced disulfide - where further, and heavily, inhibited by heavy metals; this effect was particularly evident with Zn^++^ and Cu^++^, thus confirming that reduced G6PDH is more susceptible to NADP^+^(Mg^++^ -stabilized) binding (Fig. 4).

## Discussion

The recombinant plastidic isoform *Pt*P2-G6PDH WT showed kinetic properties resembling those observed for the recombinant plastidic G6PDH in potato^13^, P2-G6PDH purified from barley roots^12^, and recombinant barley *Hv*P2-G6PDH, with and without the his tag^20^. Furthermore, *Pt*P2-G6PDH preparations utilized in this study exhibited coincident properties with those previously described^21^.

Although most of the kinetic properties of G6PDH have been studied by a number of sources, a detailed analysis of Mg^++^ effects on plant plastidic G6PDH is still lacking. In plants, ATP can inhibit G6PDH for the competition of nucleotides triphosphate with respect to Mg^++^ availability^12^. Therefore, we investigated the requirement of Mg^++^ by *Pt*P2-G6PDH activity.

The measurement of dependence of *Pt*P2-G6PDH activity on Mg^++^ resulted in a Km_Mg_^++^ of 76 μM; at this regard, it should be underlined that during standard assays the concentration of Mg^++^ is 5 mM (over 60-fold Km_Mg_^++^). Most important, Mg^++^ levels in the cells generally exceeds 1 mM (15-fold Km value), therefore under physiological conditions Mg^++^ is not limiting G6PDH reaction.

In Brenda database (www.brenda-enzymes.org)^41,42^, it is possible to verify that many studies describe the effects of metals on G6PDH activity; just limiting these to the HMs utilized in this study, it can be noted that Pb^++^ modifies the kinetic properties of the enzyme in fishes; Cd^++^ severely affects G6PDH activity in Bacteria, Fungi, Vertebrates; Ni^++^ affects the kinetic properties of the enzyme in mammals; Zn^++^ has severe effects on G6PDH from a number of sources; Cu^++^ has severe effects on G6PDH from bacteria and animals; (Supplementary Table S3, and references therein).

Thus, the inhibiting effects of different HMs on G6PDH, and the possible competition with Mg^++^ were investigated using recombinant *Pt*P2-G6PDH: different effects were observed depending on element, concentration and exposition time.

Pb^++^ was ineffective, being reaction rate over 95% of the initial value even after 1 h at 2 mM Pb^++^. The cofactor binding site is stable and shielded, thus resulting in protected access from at least Pb^++^, indicating a scarce ability of Pb^++^ to be a competitor of Mg^++^, and to bind NADP^+^. Thus, Pb^++^ did not sensibly inhibit G6PDH activity.

Cd^++^ and Ni^++^ showed immediate poisoning effects, scarcely depending on their concentration, suggesting that these elements are able to inhibit G6PDH activity possibly disrupting enzyme functional structure.

In contrast, physiological cations as Cu^++^ resulted in a reversible inhibition of enzymatic activities.

These results clearly suggest that these elements compete with binding sites on the protein, and it could be argued that the physiological competitor could be Mg^++^.

The calculation of IC_50_ for each element confirmed that Cu^++^ as the most effective, about 5-fold more than Cd^++^, and 100-fold more than Pb^++^.

Therefore, in order to clarify the type of effect exerted by metals on G6PDH, kinetic competition experiments were made with Cu^++^ and Cd^++^, using sub-saturation levels of Mg^++^. These results unequivocally confirm Cu^++^ as a competitive inhibitor of Mg^++^ for activity, while Cd^++^ exerted a mixed type inhibition.

On the other hand, a comparison between the stronger effects of Cu^++^ on Mg^++^-bound enzyme with respect to those observed on the enzyme deprived of Mg^++^, suggests that the inhibition exerted by Cu^++^ vs Mg^++^ could be not purely competitive, and possibly indicate a partially mixed effect. It could be argued that, even if Cu^++^ may compete with Mg^++^ for specific binding sites, a further inhibition can be exerted on the enzyme when it is fully stabilized Mg^++^. This effect could be caused by interferences of Cu^++^ with cystine bridges stabilized by Magnesium in the functional enzyme.

It should be noted that Cu^++^ is able to form more stable complexes with proteins than Mg^++^; therefore, the cell machinery regulates the levels of the competing metals, in order to favor the weaker cation (e.g. Mg^++^) hence improving its attractiveness for the target enzyme^40^: our data confirm that this would be true at least for Cu^++^ in *Pt*P2-G6PDH.

Intriguingly, Ki_Cu_^++^ is 20-fold higher than Km_Mg_^++^, suggesting that Cu^++^ could be a strong competitor of Mg^++^, if its intracellular levels were not maintained low. On the other hand, Ki_Cd_^++^ is two-fold Km_Mg_^++^ value, indicating that this metal possibly is able to strongly modify the functional enzyme structure.

Dudev and Lim^40^ demonstrated that cysteine residues exhibit a strong preference for the Cu^++^; furthermore, between two cations exhibiting the same charge and similar R_ion_ values, the metal ion that is a better electron acceptor binds more favorably to the ligand. Thus, although Mg^++^ has the same charge and similar ionic radii of Zn^++^ and Cu^++^, the latter (Ev 1.65 and 1.90, respectively) are better charge acceptors with respect to Mg^++^ (Ev 1.31); as consequence, Cu^++^ and Zn^++^ complexes are more favorable and stable than corresponding Mg^++^ complex with the same ligands. This would further confirm the need of consistently higher concentrations of Mg^++^ with respect to Zn^++^ and Cu^++^ in plant cells.

Magnesium is the most abundant metal di-cation in cells, and the affinity of Mg^++^ binding sites vs Mg^++^ itself is not particularly high^40^. On the other hand, Cu^++^ substitution of Mg^++^ may represent a heavy poisoning for enzymatic proteins, due to the capability of Cu^++^ to form strong binding with amino acid residues, and its properties in redox reactions. Therefore, in order to avoid these detrimental effects, the intracellular concentrations of Cu^++^ and Zn^++^ are maintained very low (10^−8^ or less), even if both these elements are essential nutrients; on the other hand, cell physiology maintains Mg^++^ concentration very high (in the order of 10^−3^ M). Under polluting conditions, it cannot be excluded that competing metals, such as Cu^++^, would be able to bind to Mg^++^-sites, severely poisoning cells.

Under this light, the mechanisms regulating the relative levels of these nutrients appear intriguingly important for the modulation of basal metabolism in living cells.

On the other hand, it should be remembered that all plant plastidic G6PDH isoforms are regulated by thioredoxins^21,26,43^: the reduction of a disulfide formed by two cysteines in the N-terminus of the protein resulted in a severe decrease in enzymatic rate^21,27,44^. The formation of this disulfide has been suggested to result in an improved stability of G6PDH causing an increased enzymatic activity^21,26^. Up to now, G6PDH crystals had been obtained only for some microorganisms^45,46^ and human^47^ enzymes, and all these enzymes are cytosolic, isoforms lacking of the regulatory disulfide, typical of organelle-located isoforms^44,48^.

Thus, we choose to utilize cysteine-mutagenized proteins (cysteine to serine) to investigate the possible role of cysteine residues in the stability and modulation of plant plastidic G6PDH by HMs.

The unspecific inhibition by HMs observed in C194S and C242S mutants would sustain previous results^21^, confirming that these two cysteine residues play a role in the binding of the NADPH as inhibitor, modulating its physiological role^23,49^. Particularly, C194 is not relevant for redox regulation in P2-G6PDH from potato (where *Pt*C194 correspond to C168)^50^. It could be supposed that the HMs would further reduce the accessibility of NADPH to the enzyme by both substituting Mg^++^ in the stabilization of the reduced cofactor, and possibly occupying Mg^++^ binding site(s) in the functional P2-G6PDH structure.

These hypotheses are confirmed by the results observed in C145S mutant showing a behavior similar to WT enzyme. This residue has been previously indicated as located close to NADP^+^ binding site and possibly influencing cofactor accessibility, but not involved directly in activity regulation^21^.

C175S and C183S are enzymes mutagenized in the regulatory cysteines; intriguingly, their activities, about 20% of WT, where further, and heavily, inhibited by metals, and particularly by Zn^++^ and Cu^++^, up to 1% if the V_max_ measured in the full activated WT enzyme.

This could open interesting scenarios for G6PDH regulation: the enzyme would be controlled by NADP^+^ (Mg^++^ - bound)^12^, stabilizing the enzymatic structure even under reducing conditions (e.g. light), when increased reductants are requested^2^; in the dark, lower levels of reductants - and increased Trx_ox_ - would activate the enzyme^26^, (it can be argued, by forming the disulfide bridge), finally turning on the oxidative pentose phosphate pathway.

C175S and C183S G6PDHs represent “reduced forms” of P2-G6PDH, showing about 20% of control activity; HMs possibly occupy the Mg^++^ binding site(s), lowering the residual enzymatic activity to zero.

Therefore, we advantaged by bioinformatics tools^51^, and using human cytosolic G6PDH^52^ as template, defining a putative 3D structure of *Pt*P2-G6PDH, based on known cytosolic *Hs*G6PDH 3D crystallographic structure. The template identity with human *Hs*G6PDH is provided in Supplementary Table S2.

**Figure 5:**
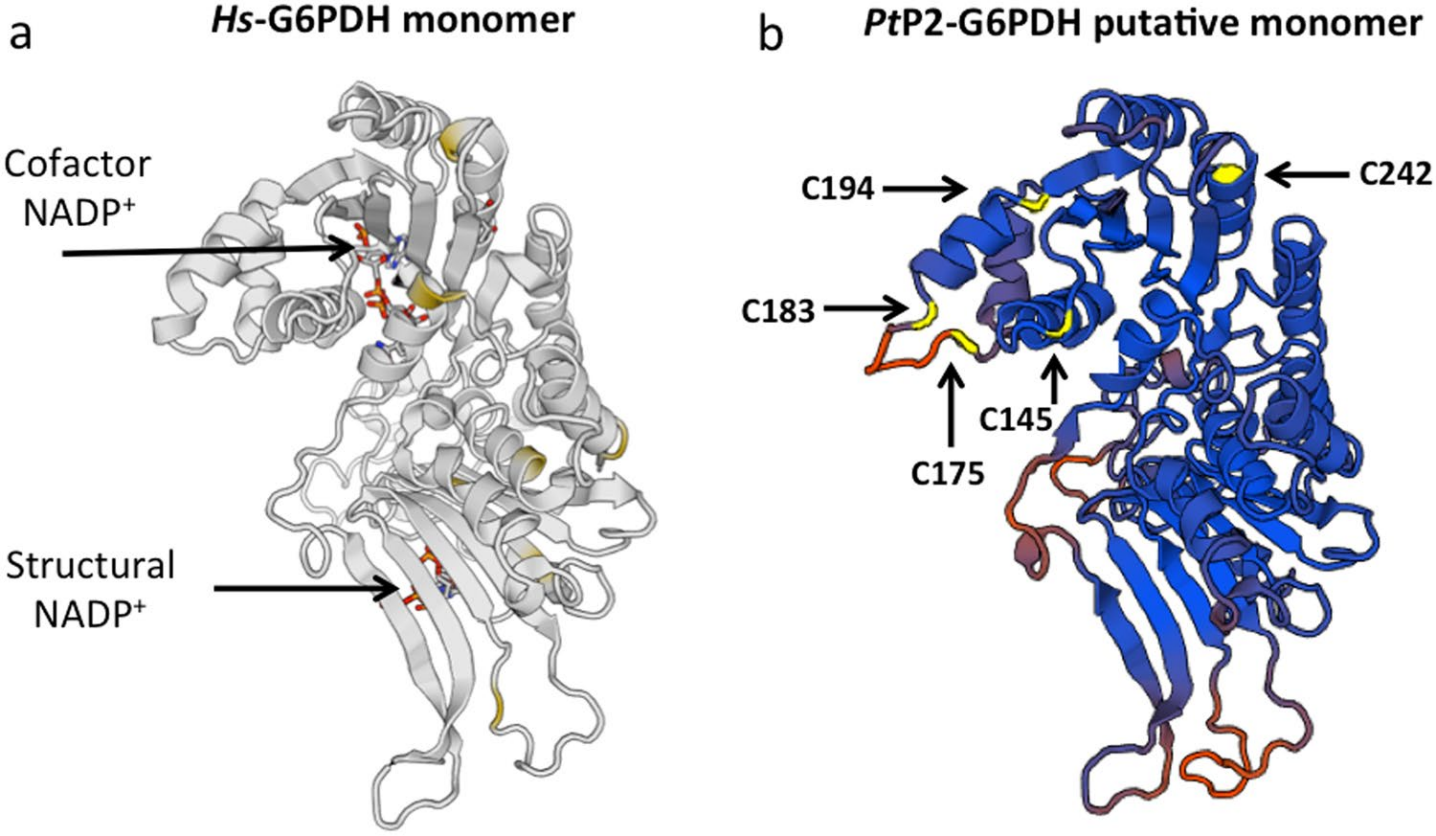
Determination of theoretical structure of *Pt*P2-G6PDH WT. (**a**) Crystallographic structure of human cytosolic *Hs*G6PDH^36,52^ harboring cofactor NADP^+^ and structural stabilizing NADP^+^ for comparison with (**b**), putative structure of plastidic *Pt*P2-G6PDH showing the positions occupied by cysteine residues (yellow). In (**a**) the not-conserved cysteines present in the *Hs*G6PDH are in pale yellow. Details for monomers design are provided in the text, and Supplementary Tables 1 and 3.

In Fig. 5a the *Hs*G6PDH monomer is shown, to better evidence the similarities with *Pt*P2-G6PDH putative structure designed on this model; the positions occupied by cysteine residues in the plant enzyme are evidenced. Despite the obvious similarities, the main difference is in the unstructured loop on the N-terminal where *Pt*P2-G6PDH exposes the two cysteines (C175 – C183) involved in the regulatory disulfide bond^21^. Even if 3D crystals of plastidic G6PDH are still not available, it has been suggested that disulfide formation within this loop induces structural change in NADP^+^ cofactor binding and may facilitate the access of cofactor to the active site^26^. The C145 residue is located near to the active site, and NADP^+^ binding. The other two residues, C194 and C242, are on the exposed part of the enzyme, and possibly would influence the harboring of NADPH. Mutagenized isoforms in Cys^145^, Cys^194^ and Cys^242^ - residues not involved in the disulfide - show an unspecific and similar inhibition by HMs. Therefore, it can be argued that the presence of Mg^++^ close to the active site is crucial for the access of NADP^+^ to the active site, even in the “reduced” enzyme: when different physiological cations (or highly soluble, such as Cd^++^) are present, a strongly reduced accessibility of the cofactor to the active site occurs, totally inhibiting the enzymatic reaction.

In conclusion, the data here presented strongly support the hypothesis that NADP^+^ - Mg^++^ levels in the plastid are able to modulate G6PDH activity; from this point of view, the competition between HMs and Mg^++^ can be critical and possibly utilized both to investigate the regulation of plant plastidic G6PDH, and the role played by functional cations. Furthermore, the role of the disulfide bridge in the N terminus has been confirmed to be pivotal in the modulation of activity, possibly playing critical roles in the stabilization of the active enzyme.

## Methods

### Overexpression and purification of recombinant PtP2-G6PDH

The plastidic G6PDH sequence encoding for *Pt*P2-G6PDH WT was identified from *Populus trichocarpa* genome (http://www.plantgdb.org/PtGDB/) and sequenced using cDNA (courtesy of J-P. Jacquot and N. Rouhier, Universitè de Lorraine, Nancy, France). The sequence was cloned in pET15b plasmid and recombinant protein was overexpressed in *E. coli* strain BL21(DE3)psBET as standard host^21^. The cells were disrupted by sonication and after centrifugation, the supernatant, containing most of the enzyme activity, was utilized for purification using Sephadex G25 gel filtration and Immobilized Metal Ion Affinity Chromatography (IMAC) with Ni^++^ and imidazole elution^20,21^.

Mutagenized *Pt*P2-G6PDHs were obtained using two complementary mutagenic primers, by substituting the 5 cysteine residues present in the active enzyme sequence into serine as described elsewhere^21^. The recombinant, mutagenized enzymes were purified as for the *Pt*P2-G6PDH WT.

### G6PDH activity determination

G6PDH activity assays were run at 25 °C by measuring the reduction of NADP^+^ to NADPH at 340 nm by G6PDH in the presence of glucose-6-phosphate (G6P) in a 1 cm cuvette in a spectrophotometer Cary 60 (Agilent Technologies)^20^. Assays were always carried out in duplicates.

The reaction mixture (final volume 1 ml) contained 5 mM MgCl_2_, 150 μM NADP^+^, and 3 mM G6P in 30 mM Tris–HCl buffer, pH 7.5; 2–10 μl of purified enzyme (1 to 5 mg prot. ml^−1^) were utilized; blank without G6P.

One unit of enzyme (U) activity defined as the amount of enzyme that reduced 1 μmol NADP^+^ per minute, the total activity was expressed as units per mg of protein.

The determination of protein concentrations was according to Bradford (1976) using bovine serum albumin (BSA) as standard protein.

### Electrophoresis and western blotting analysis

SDS-PAGE as previously described^21^ performed using a 10% polyacrylamide resolving gel with a 4% stacking gel (Bio-Rad Mini-Protean). Western blotting, made using primary potato antibodies for P1-G6PDH, P2-G6PDH and Cy-G6PDH isoforms^13^ and Anti-His6 (Roche). Potato antisera were previously proven to react with and discriminate the different G6PDH isoforms^20,21,53^. After incubating the membrane with secondary antibodies and cross-reacting polypeptides stained by enhanced chemiluminescence.

### *In vitro* effects of metal ions

Heavy metals were tested for their effects on purified recombinant *Pt*P2G6PDH. The salts of HM were CdCl_2_, CuSO_4_, ZnSO_4_, NiSO_4_, Pb(NO_3_)_2_.

Assays were carried out using 2–10 μl of purified enzyme (1 to 5 mg prot. ml^−1^) in 30 mM Tris-HCl buffer pH 7.5, 150 μM NADP^+^, 5 mM MgCl_2_ and 3 mM G6P (final volume 1 ml), at 25 °C under standard conditions with varying concentration of metal ions.

Due to the possible inhibition effects caused by sulfates on G6PDH activity previously reported^3,54^, HM incubation assays were repeated by exposing recombinant *Pt*P2-G6PDH to 0.1, 0.5, 1 mM CuCl_2_, ZnCl_2_ and NiCl_2_ and corresponding sulfate salts for 1, 10 and 60 min (Supplementary Fig. S2).

Results were expressed as percentage of the control (activity measured without HM). All assays were performed in triplicates at each concentration used.

Metal ions concentrations that produced 50% inhibition (IC_50_) of enzymatic activity were calculated from the non-linear regression graphs.

### G6PDH alkylation

Purified *Pt*P2-G6PDH was alkylated using iodoacetic acid^55^. Briefly, 100 μl of purified enzyme (1.2 mg/ml; 20 ng prot.) were incubated with excess of iodoacetic acid (100 μM in deionized water) overnight at 20 °C in the dark. The enzyme was desalted using a Sephadex G25 column (1 ml) using an AKTA prime plus system (GE Healthcare), at 0.5 ml min^−1^ flux. As comparison, purified *Pt*P2-G6PDH was submitted to the same procedure without iodacetic acid (using the same volume as deionized water) and tested for activity as control.

### Kinetic properties of *Pt*P2-G6PDH

Affinity and inhibition constants were calculated as described previously^21,24^. Each kinetic property presented was representative of at least three different preparations; the values were calculated using GraphPad Prism software, within 95% confidence.

### Design of putative *Pt*P2-G6PDH structure

The putative structure of *Pt*P2-G6PDH was designed using Swiss-Prot model building software^51^ ProMod3 Version 1.1.0 (https://swissmodel.expasy.org).

The 3D protein structure of human cytosolic *Hs*G6PDH^47^ was utilized as template, and primary amino acid sequence of *Pt*P2-G6PDH submitted for model design. The submitted primary amino acid sequence is given in Supplementary Table S1.

The results for the homology modelling of *Pt*P2-G6PDH were submitted to SWISS-MODEL workspace. The SWISS-MODEL template library (SMTL) was searched with Blast and HHBlits for evolutionary related structures matching the target sequence. Coordinates which are conserved between the target and the template are copied from the template to the model. Insertions and deletions are remodeled using a fragment library. Side chains are then rebuilt. Finally, the geometry of the resulting model is regularized by using a force field. Report of theoretical models utilized for putative 3D modelling of *Pt*P2-G6PDH are in Supplementary Table S3.

### Chemicals

Glucose-6-phosphate (G6P), NADP^+^, and all other chemicals used were of analytical grade and purchased from Sigma-Aldrich Chemical Co., MO, USA, unless otherwise specified. Chromatographic IMAC columns were from GE Healthcare. The HMs salts (both chloride and sulfate) were furnished by Sigma Aldrich; NiSO_4_ was purchased from Carlo Erba (Milan, Italy).

## Electronic supplementary material

Supplementary Figure S1

Supplementary Figure S2

Supplementary Figure S3

Supplementary Figure S4

Supplementary Figure S5

Supplementary Table S1

Supplementary Table S2

Supplementary Table S3

Supplementary Table S4

## Data Availability

All data generated or analyzed during this study are included in this published article (and its Supplementary Information files).
