# Supplementary material for: Mechanism(s) of action of heavy metals to investigate the regulation of plastidic glucose-6-phosphate dehydrogenase
Source: Sci Rep. 2018 Sep 7;8:13481. doi: 10.1038/s41598-018-31348-y (PMC6128849; doi:10.1038/s41598-018-31348-y)
Supplement: Supplementary file 1 — Supplementary Figure S1 [file 41598_2018_31348_MOESM1_ESM.pdf]

Mechanism(s) of action of heavy metals to investigate the regulation of plastidic glucose-6-phosphate dehydrogenase

Alessia DE LILLO, Manuela CARDI, Simone LANDI, Sergio ESPOSITO*

* sergio.esposito@unina.it

Supplementary Information

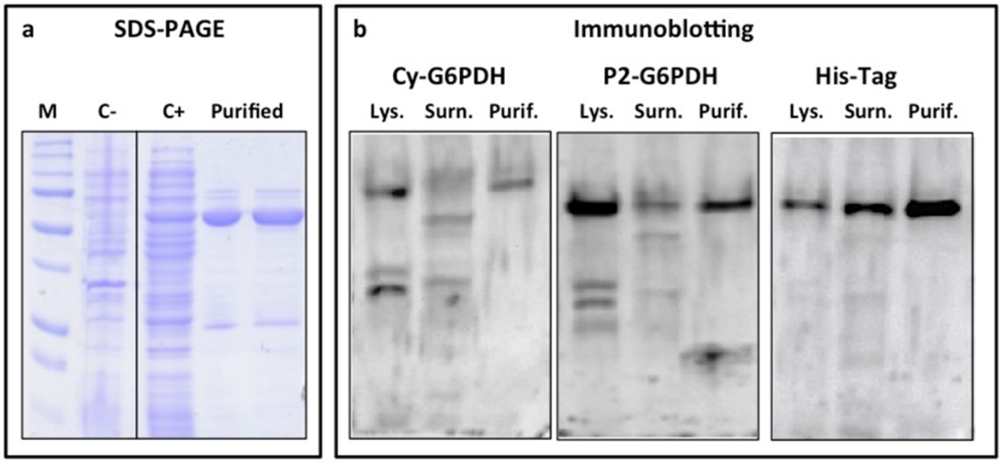

Supplementary Figure S1. Electrophoretic analysis and immunoblots of *Pt*P2-G6PDH WT.

(a) Coomassie-stained SDS gels showing a protein of the expected MW corresponding to *Pt*P2-G6PDH WT. M, Markers; C-, Control (*E. coli* without transformed plasmid); C+, transformed colonies after induction with IPTG; Purified, purified protein after IMAC step, 5µg and 10 µg protein.

(b) Immunoblotting using antisera versus Cy-G6PDH antiserum (potato[14]); P2-G6PDH antiserum (potato[14]); His_6-tag antiserum (Roche). Legend for the lanes: Lys., bacterial lysate; Surn., surnatant or soluble fraction; Purif., purified protein as in (a).
