# Supplementary material for: Mechanism(s) of action of heavy metals to investigate the regulation of plastidic glucose-6-phosphate dehydrogenase
Source: Sci Rep. 2018 Sep 7;8:13481. doi: 10.1038/s41598-018-31348-y (PMC6128849; doi:10.1038/s41598-018-31348-y)
Supplement: Supplementary file 2 — Supplementary Figure S2 [file 41598_2018_31348_MOESM2_ESM.pdf]

Mechanism(s) of action of heavy metals to investigate the regulation of plastidic glucose-6-phosphate dehydrogenase

Alessia DE LILLO, Manuela CARDI, Simone LANDI, Sergio ESPOSITO*

* sergio.esposito@unina.it

Supplementary Information

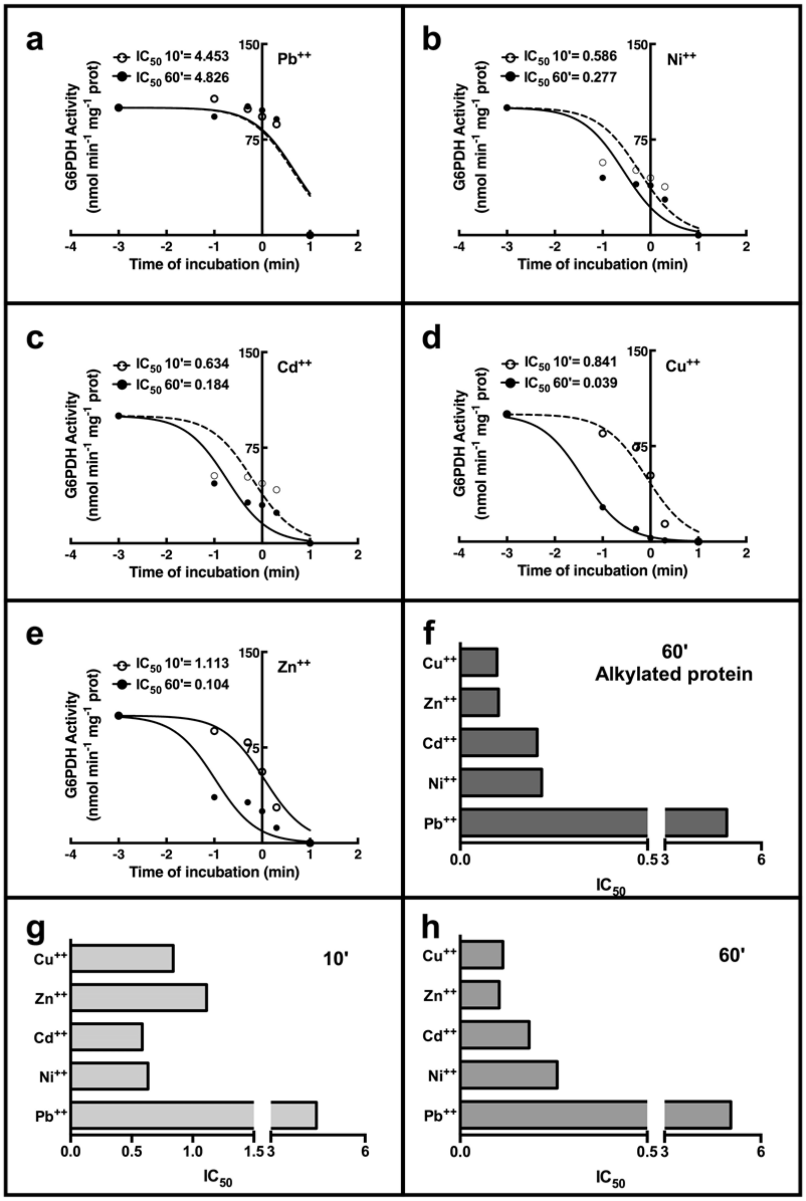

Supplementary Figure S2. Determination of IC_{50} values (mM) calculated after 10’ (open circles, dotted line) and 60’ (close circles, solid line) of incubation with different HMs related to *Pt*P2-G6PDH WT activity. (a) Pb^{++}; (b) Ni^{++}; (c) Cd^{++}; (d) Cu^{++}; (e) Zn^{++}; (f) IC_{50} values (mM) after 60’ of incubation with each metal, as calculated on alkylated enzyme to assess the influence of his-tag on HM binding; (g) and (h), IC_{50} values (mM) for each metal as calculated from the graphs (a) to (e) on his-tagged *Pt*P2-G6PDH, after 10’ and 60’ of incubation, respectively. R^2 for IC50 was between 70 and 98% (not shown). Non-linear regressions and IC_{50} values were calculated by Graph-Pad Prism software.
