# Supplementary material for: Mechanism(s) of action of heavy metals to investigate the regulation of plastidic glucose-6-phosphate dehydrogenase
Source: Sci Rep. 2018 Sep 7;8:13481. doi: 10.1038/s41598-018-31348-y (PMC6128849; doi:10.1038/s41598-018-31348-y)
Supplement: Supplementary file 3 — Supplementary Figure S3 [file 41598_2018_31348_MOESM3_ESM.pdf]

Mechanism(s) of action of heavy metals to investigate the regulation of plastidic glucose-6-phosphate dehydrogenase

Alessia DE LILLO, Manuela CARDI, Simone LANDI, Sergio ESPOSITO*

* sergio.esposito@unina.it

Supplementary Information

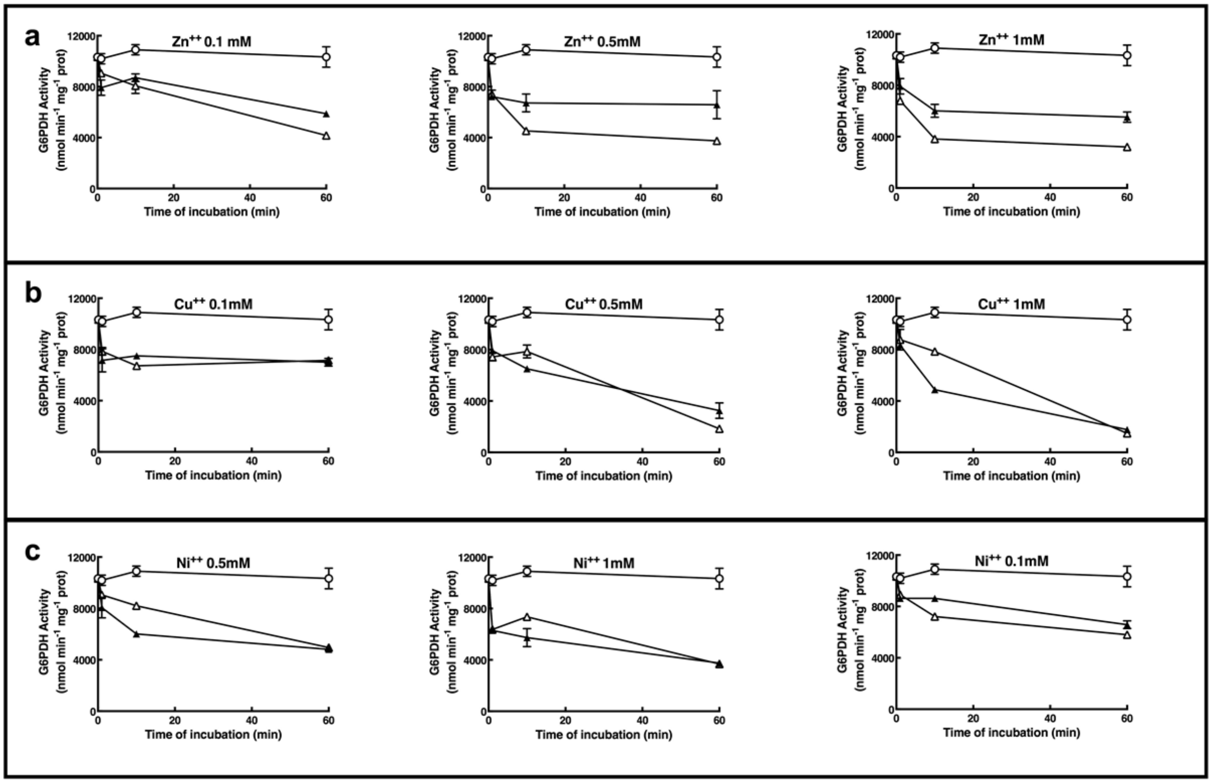

Supplementary Figure S3. Comparison between the effects of chloride and sulphate salts of heavy metals on recombinant *Pt*P2-G6PDH WT activity.

Purified enzyme was incubated without heavy metals (○); or in the presence of different metals chloride (▲) and sulfate (△) salts at increasing concentrations: 0.1mM (left); 0.5mM (center), 1mM (right) for 1, 10, 60 min. (a) Zinc (Zn^{++}); (b) Copper (Cu^{++}); (c) Nickel (Ni^{++}).
