# Supplementary material for: Mechanism(s) of action of heavy metals to investigate the regulation of plastidic glucose-6-phosphate dehydrogenase
Source: Sci Rep. 2018 Sep 7;8:13481. doi: 10.1038/s41598-018-31348-y (PMC6128849; doi:10.1038/s41598-018-31348-y)
Supplement: Supplementary file 4 — Supplementary Figure S4 [file 41598_2018_31348_MOESM4_ESM.pdf]

Mechanism(s) of action of heavy metals to investigate the regulation of plastidic glucose-6-phosphate dehydrogenase

Alessia DE LILLO, Manuela CARDI, Simone LANDI, Sergio ESPOSITO*

* sergio.esposito@unina.it

Supplementary Information

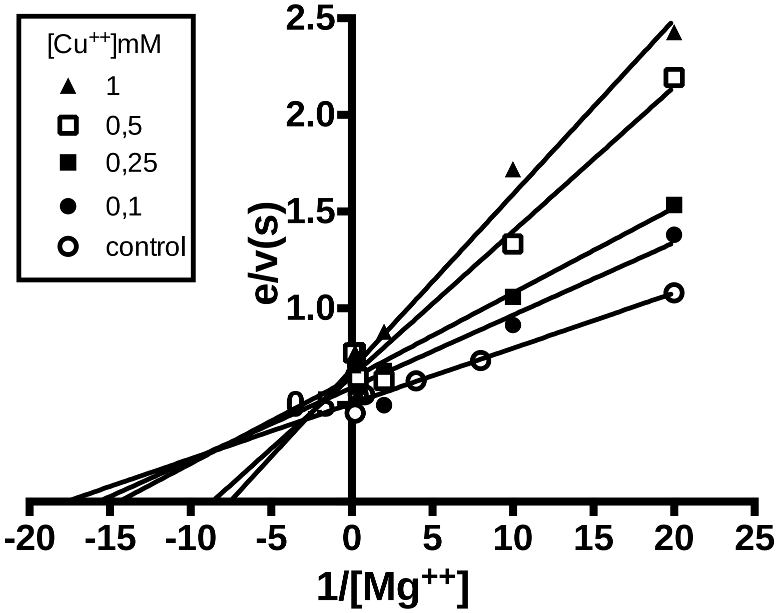

Supplementary Figure S4. Double reciprocal plot of competition experiment measuring Km_{Mg}^{++} in the presence of increasing levels of Cu^{++} on *Pt*P2-G6PDH.

Purified enzyme was desalted on Sephadex G25 twice to obtain a no-Mg^{++} enzyme. Then this preparation was tested to determine Km_{Mg}^{++}= 0.060 mM (V_{max}: 1.9 mM). When desalted (no-Mg^{++}) enzyme was exposed to Cu^{++} levels from 0 to 1mM (as indicated in the in-figure legend), Kms_{Mg}^{++} increased up to 0.15mM; V_{max} remained substantially unchanged, 1.68 ± 0.16mM.

Copper was provided as $CuCl_2$ to avoid possible sulphate inhibition.

The regressions were calculated by Graphpad Prism software within 91-98% confidence.
