# Supplementary material for: Mechanism(s) of action of heavy metals to investigate the regulation of plastidic glucose-6-phosphate dehydrogenase
Source: Sci Rep. 2018 Sep 7;8:13481. doi: 10.1038/s41598-018-31348-y (PMC6128849; doi:10.1038/s41598-018-31348-y)
Supplement: Supplementary file 5 — Supplementary Figure S5 [file 41598_2018_31348_MOESM5_ESM.pdf]

Mechanism(s) of action of heavy metals to investigate the regulation of plastidic glucose-6-phosphate dehydrogenase

Alessia DE LILLO, Manuela CARDI, Simone LANDI, Sergio ESPOSITO*

* sergio.esposito@unina.it

Supplementary Information

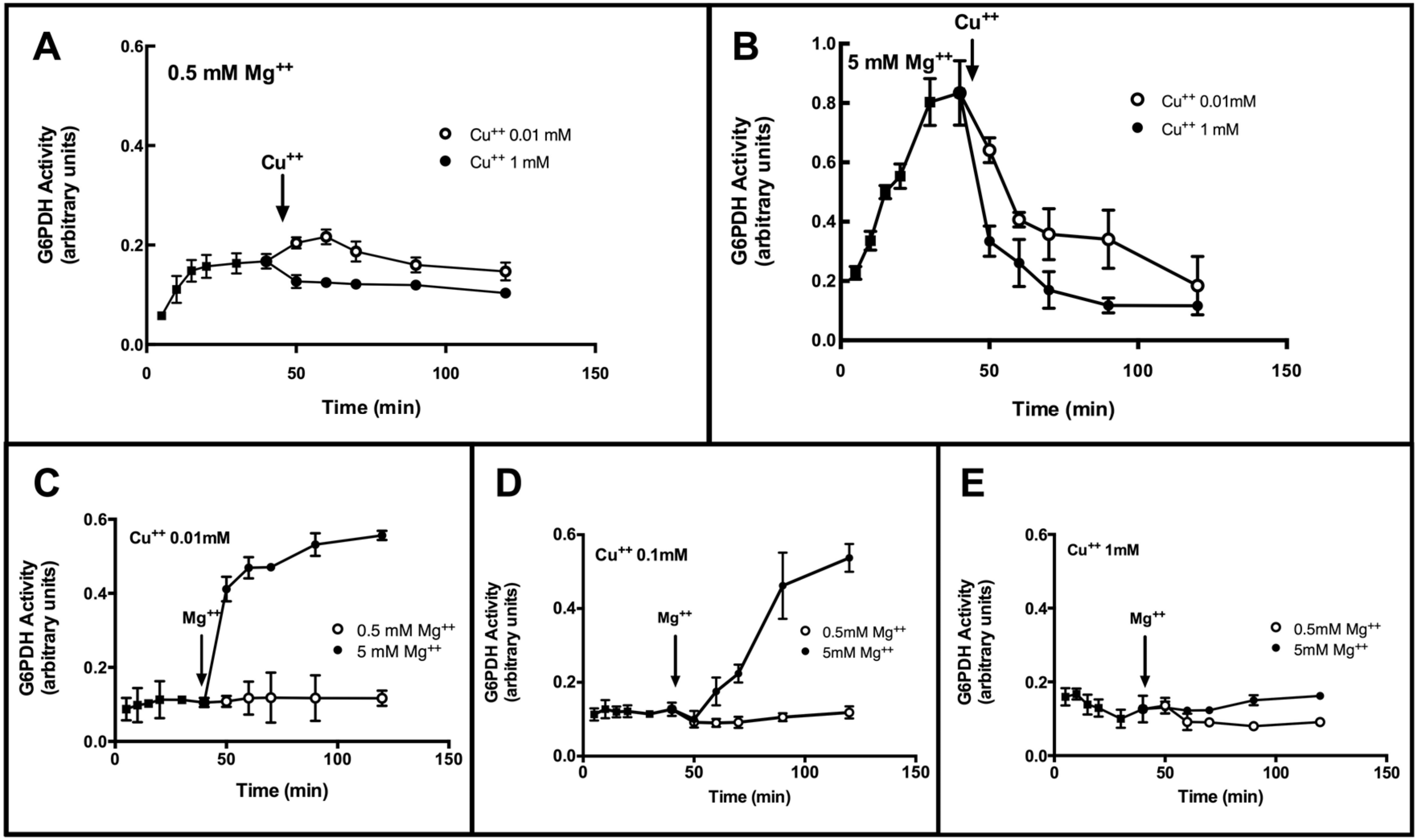
A
0.5 mM Mg++
Cu++
Cu++ 0.01 mM
Cu++ 1 mM
G6PDH Activity (arbitrary units)
Time (min)
B
5 mM Mg++
Cu++
Cu++ 0.01mM
Cu++ 1 mM
G6PDH Activity (arbitrary units)
Time (min)
C
Cu++ 0.01mM
Mg++
0.5 mM Mg++
5 mM Mg++
G6PDH Activity (arbitrary units)
Time (min)
D
Cu++ 0.1mM
Mg++
0.5mM Mg++
5mM Mg++
G6PDH Activity (arbitrary units)
Time (min)
E
Cu++ 1mM
Mg++
0.5mM Mg++
5mM Mg++
G6PDH Activity (arbitrary units)
Time (min)

Supplementary Figure S5. Tests for reversibility of Cu^{++} inhibition of *Pt*P2-G6PDH activity. Purified enzyme was desalted on Sephadex G25 twice to obtain an enzyme containing no Mg^{++}. Then, this preparation was tested for G6PDH activity in assay mixture in the presence of 0.5mM Mg^{++} (A); or 5mM Mg^{++} (B). After 45min, when enzyme activity was stable, 0.01mM or 1mM Cu^{++} were added to the assay mix and the inhibition of the activity measured.

In the second set of measurements, the enzyme containing no Mg^{++} was tested for G6PDH activity in assay mixture in the presence of 0.01mM (C); 0.1mM (D), and 1mM (E) Cu^{++} after 45min, when enzyme activity was stable, 0.5mM Mg^{++} or 5mM Mg^{++} were added to the assay mix and the re-activation of activity measured.

Copper was provided as $CuCl_2$ to avoid possible sulphate inhibition. The error bars indicate standard error from at least three different measurements.
