# Supplementary material for: Mechanism(s) of action of heavy metals to investigate the regulation of plastidic glucose-6-phosphate dehydrogenase
Source: Sci Rep. 2018 Sep 7;8:13481. doi: 10.1038/s41598-018-31348-y (PMC6128849; doi:10.1038/s41598-018-31348-y)
Supplement: Supplementary file 6 — Supplementary Table S1 [file 41598_2018_31348_MOESM6_ESM.pdf]

Mechanism(s) of action of heavy metals to investigate the regulation of plastidic glucose-6-phosphate dehydrogenase

Alessia DE LILLO, Manuela CARDI, Simone LANDI, Sergio ESPOSITO*

* sergio.esposito@unina.it

Supplementary Information

	**Km_{G6P} (mM)**	**Km_{NADP^+} (µM)**	**Ki_{NADPH} (µM)**	**V_{max} ($U \cdot mg^{-1}$ prot)**	**Monomer MW His-tagged (kDa)**
*Pt*P2-G6PDH WT	0.95± 0.12	28 ± 4	73 ± .3	7800	**64**
*Pt*P2-G6PDH WT*	1.06 ± 0.06	15 ± 3	74.1 ± 2.5	8000	**61**

Supplementary Table S1 - Kinetic properties of wild type recombinant G6PDH from *Populus trichocarpa* (*Pt*P2-G6PDH WT). In the first line, the main kinetic properties measured in purified protein preparations used in this study; in the second line the previously published parameters for the same enzyme[22]. Molecular weights were calculated by using the relative mobility factor method in SDS-PAGE (gels and immunoblotting are shown in Supplementary Figure S1).
