# Supplementary material for: Mechanism(s) of action of heavy metals to investigate the regulation of plastidic glucose-6-phosphate dehydrogenase
Source: Sci Rep. 2018 Sep 7;8:13481. doi: 10.1038/s41598-018-31348-y (PMC6128849; doi:10.1038/s41598-018-31348-y)
Supplement: Supplementary file 7 — Supplementary Table S2 [file 41598_2018_31348_MOESM7_ESM.pdf]

Mechanism(s) of action of heavy metals to investigate the regulation of plastidic glucose-6-phosphate dehydrogenase

Alessia DE LILLO, Manuela CARDI, Simone LANDI, Sergio ESPOSITO*

* sergio.esposito@unina.it

Supplementary Information

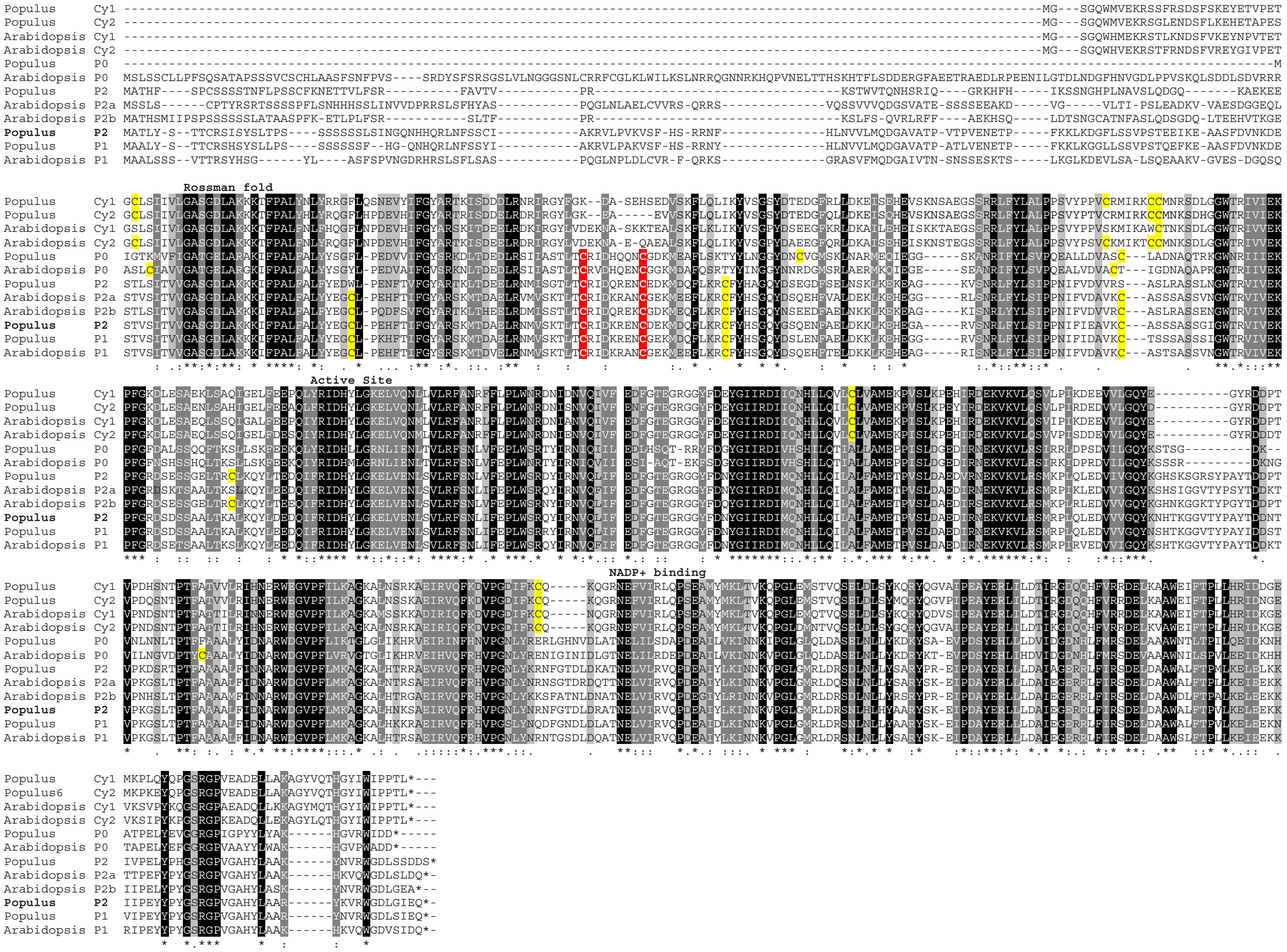
Rossman fold
Active Site
NADP+ binding

Supplementary Table S2. Comparison among aminoacidic sequences of G6PDH isoforms from *Populus* and *Arabidopsis*. Legend: CY, cytosolic isoform; P0, peroxisome/cytosolic/plastidial isoform; P1, chloroplastic isoform; P2, plastidial isoform. Typical motifs are indicated: Rossman-fold; active site, and $NADP^+$ binding site. Cysteine residues present in the sequences are highlighted in yellow. Regulatory cysteines involved in the formation of the disulfide are in white letters highlighted in red. Conservation of residues in the different sequences is indicated in grey scale from white (no conservation) to black (same residue in all isoforms).

List of sequences: *Populus* CYa, estExt_Genewise1_v1.C_LG_XVII0625; *Populus* CYb, grail3.0054015801; *Arabidopsis* CYa At3g27300.1; *Arabidopsis* CYb At5g40760.1; *Populus* P0, estExt_fgenesh4_pm.C_LG_XIII0018; *Arabidopsis* P0, At1g09420.1; *Populus* P1, fgenesh4_pm.C_LG_XIV000487; *Arabidopsis* P1, At5g13110.1; *Arabidopsis* P2a, At1g24280.1; *Arabidopsis* P2b, At5g35790.1; *Populus* P2a, estExt_Genewise1_v1.C_LG_I7789; *Populus* P2b, eugene3.0003137
