# Supplementary material for: Mechanism(s) of action of heavy metals to investigate the regulation of plastidic glucose-6-phosphate dehydrogenase
Source: Sci Rep. 2018 Sep 7;8:13481. doi: 10.1038/s41598-018-31348-y (PMC6128849; doi:10.1038/s41598-018-31348-y)
Supplement: Supplementary file 8 — Supplementary Table S3 [file 41598_2018_31348_MOESM8_ESM.pdf]

Mechanism(s) of action of heavy metals to investigate the regulation of plastidic glucose-6-phosphate dehydrogenase

Alessia DE LILLO, Manuela CARDI, Simone LANDI, Sergio ESPOSITO*

* sergio.esposito@unina.it

Supplementary Information

Supplementary Table S3. Report of theoretical models utilised in this work for putative 3D modelling of *Pt*P2-G6PDH WT.

*Pt*P2-G6PDH WT sequence was utilised to build the putative 3D structure based on human cytosolic *Hs*G6PDH[40]. The template's quality has been predicted from features of the target-template alignment. The templates with the highest quality have then been selected for model building.

Models are built based on the target-template alignment using ProMod3.

	**Template**	**coverage**	**Sequence Identity**	**Sequence similarity**	**GMQE**	**QMEAN**
**Monomer** (*requested by user*)	5aq1.1.A	0.79	46.40	0.42	0.65	-1.73
**Homo-dimer**	2bh9.1A	0.79	48.31	0.43	0.66	-1.03
**Homo-tetramer $NADP^+$ bound**	5aq1.1.A	0.79	46.40	0.42	0.65	-1.30
**Homo-tetramer $NADP^+$-G6P bound**	5aq1.1.A	0.75	49. 10	0.43	0.63	-1.72
