# Supplementary material for: Mechanism(s) of action of heavy metals to investigate the regulation of plastidic glucose-6-phosphate dehydrogenase
Source: Sci Rep. 2018 Sep 7;8:13481. doi: 10.1038/s41598-018-31348-y (PMC6128849; doi:10.1038/s41598-018-31348-y)
Supplement: Supplementary file 9 — Supplementary Table S4 [file 41598_2018_31348_MOESM9_ESM.pdf]

Mechanism(s) of action of heavy metals to investigate the regulation of plastidic glucose-6-phosphate dehydrogenase

Alessia DE LILLO, Manuela CARDI, Simone LANDI, Sergio ESPOSITO*

* sergio.esposito@unina.it

Supplemental Table S4 - Effects of heavy metals utilized in this study on G6PDH from different sources as presented in brenda database (http://www.brenda-enzymes.org/). In the third column the description of the main effects noted is provided. Where measured, changes in kinetic properties and IC_{50} values are reported in fourth column and fifth column, respectively. The original references are listed below.

Heavy metals	Organisms	Authors comments	KI	EC50	References
**Lead (Pb^{++})**	*Oncorhynchus mykiss*	noncompetitive inhibition	0.213 mM	0.78 mM	(2)
**Nickel (Ni^{++})**	*Camelus dromedarius*	95% residual activity at 2 mM			(5)
**Cadmium (Cd^{++})**	*Aspergilus paraticus*				(8)
	Cryptococcus neoformans				(9)
	Deinococcus radiophilus	53% of G6PDH1 inhibition at 1 mM			(10)
	Escherichia coli	83% of G6PDH inhibition at 1 mM			(1)
	Lactobacillus buchneri				(7)
	Oncorhynchus mykiss	noncompetitive inhibition	2.034 mM	1.97 mM	(2)
**Copper (Cu^{++})**	*Bos taurus*	65% residual activity at 2 mM (G6PD2),<br>50% residual activity at 2 mM (G6PD1)			(6)
	Brugia malayi				(11)
	Camelus dromedarius	15% residual activity at 5 mM			(5)
	Deinococcus radiophilus	24% inhibition of G6PDH-1 at 1 mM<br>78% inhibition of G6PDH-2 at 1 mM			(10)
	Escherichia coli	Complete inhibition using 1 mM of metal			(1)
	Lactobacillus buchneri				(7)
	Oncorhynchus mykiss	noncompetitive inhibition	1.721 mM	1.19 mM	(2)

**Zinc (Zn^{++})**	*Aspergilus aculeatus*	competitive, 40% inhibition at 0.01 mM	0.0066 mM		(4)
	Aspergilus paraticus				(8)
	Bos taurus	58% residual activity at 5 mM (G6PD2)<br>35% residual activity at 2 mM (G6PD1)			(6)
	Camelus dromedarius	50% residual activity at 5 mM of metal			(5)
	Cryptococcus neoformans				(9)
	Deinococcus radiophilus	83% inhibition of G6PDH-2 at 1 mM,<br>94% inhibition of G6PDH-1 at 1 mM			(10)
	Homo sapiens				(3)
	Lactobacillus buchneri				(7)
	Oncorhynchus mykiss	noncompetitive inhibition	2.77 mM	2.16 mM	(2)
	Sus scrofa				(3)

References to Supplemental Table S4

1. Banerjee, S. & Fraenkel, D.G. Glucose-6-phosphate dehydrogenase from Escherichia coli and from a 'high-level' mutant. *J. Bacteriol.* **110**, 155-160; (1972).
2. Cankaya, M., Sisecioglu, M., Ciftci, M. & Ozdemir, H. Effects of some metal ions on trout liver glucose 6-phosphate dehydrogenase. *Res. J. Environ. Toxicol.* **5**, 385-391 (2011).
3. Cho, S.W. & Joshi, J.G. Characterization of glucose-6-phosphate dehydrogenase isozymes from human and pig brain. *Neuroscience.* **38**, 819-828 (1990).
4. Ibraheem, O., Adewale, I.O. & Afolayan, A. Purification and properties of glucose 6-phosphate dehydrogenase from Aspergillus aculeatus. *J. Biochem. Mol. Biol.* **38**, 584-590 (2005).
5. Ibrahim, M., Ghazy, A., Salem, A., Ghazy, M. & Abdel-Monsef, M. Purification and characterization of glucose-6-phosphate dehydrogenase from camel liver. *Enzyme Res.* 2014, 714054 (2014).
6. Ibrahim, M.A., Ghazy, A.H., Salem, A.M., Ghazy, M.A. & Abdel-Monsef, M.M. Biochemical characterization of buffalo liver glucose-6-phosphate dehydrogenase isoforms. *Protein J.* **34**, 193-204 (2015).
7. Kawai, K. & Eguchi, Y. Properties of Lactobacillus glucose 6-phosphate dehydrogenase. *J. Ferment. Technol.* 57, 369-371; (1979).
8. Niehaus, W.G. & Dilts, R.P. Purification and characterization of glucose-6-phosphate dehydrogenase from Aspergillus parasiticus. *Arch. Biochem. Biophys.* **228**, 113-119; (1984).
9. Niehaus, W.G. & Mallett, T.C. Purification and characterization of glucose-6-phosphate dehydrogenase from Cryptococcus neoformans: identification as 'nothing dehydrogenase. *Arch. Biochem. Biophys.* **313**, 304-309; (1994).
10. Sung, J.Y. & Lee, Y.N. Isoforms of glucose 6-phosphate dehydrogenase in Deinococcus radiophilus. *J. Microbiol.* **45**, 318-325; (2007).
11. Verma, A., Suthar, M.K., Doharey, P.K., Gupta, S., Yadav, S., Chauhan, P.M. & Saxena, J.K. Molecular cloning and characterization of glucose-6-phosphate dehydrogenase from Brugia malayi. *Parasitology* **140**, 897-906 (2013).
